# The Rheumatic Diathesis

**Published:** 1912-01-20

**Authors:** 


### The Hospital

The Workers' Newspaper of Administrative Medicine and Institutional Life, Administration, National Insurance and Health.

No. 1332, Vol. LI. SATURDAY, JANUARY 20th, 1912. PRICE ONE PENNY.

### THE RHEUMATIC DIATHESIS.

Up to the last quarter of the nineteenth century the belief of the medical profession in certain "diatheses" was almost universal; and of these the rheumatic or arthritic, the strumous or scrofulous, the bilious, and the nervous were the most commonly admitted and recognised. The term diathesis implies, in the words of one who still upholds the existence of these traits, that there are inborn personal peculiarities and varieties of constitution, and that such differences are expressed by degrees of vulnerability, immunity, and proclivity. Nay, more, the signs whereby the subjects of a particular diathesis (or diatheses) may be recognised before the exhibition of any disease due to the constitutional peculiarity, were described in detail. Thus the scrofulous diathesis was held to be divisible into two subdivisions, respectively the fine tuberculous and the coarse strumous; and these two types were illustrated pictorially in leading textbooks (see Treves' "System of Surgery," 1895).

With the rise and progress of bacteriology, dating from about thirty years ago, conceptions of disease and of ætiology underwent gradual but profound modifications. No longer were physicians content to ascribe phthisis to an assumed diathesis, rendering the patient especially liable to that disease. The bacillus tuberculosis was demonstrated to be the prime cause of tuberculous lesions, and for a time attention was diverted from the patient to the microbe. The diathetic apologists adapted their hypothesis to the new era by making the scrofulous diathesis a concomitant of undue vulnerability to the bacillus of Koch. But by this time it was beginning to dawn on the profession that many who develop tuberculosis show none of the features alleged to characterise the scrofulous diathesis; and that many in whom such signs are displayed do not fall victims to tuberculosis. Accordingly, the habit of ascribing disease to a diathesis waned, and is now distinctly out of fashion.

But after a while attention was once more directed to the constitution, and not solely to the invading microbe. The opsonic index of Wright, the researches on immunity of Ehrlich and Metchnikoff, showed that the soil as well as the seed is of importance. Then came the suggestion that those signs, once supposed to characterise a proclivity to some disease (or group of diseases), are in reality the very earliest signs of the disease itself. Thus the symptoms hitherto ascribed to struma were regarded, on this new supposition, as evidences of a very slight or early invasion of disease. This bold conception explained simply enough the subsequent occurrence of fully developed tuberculosis in those of strumous or scrofulous habit; for it treated the former as merely an extension and an aggravation of an existing infection indicated by the latter. It captivated the newer generation of medical men, brought up in daily contact with the bacteriology; but it did not satisfy many of the older school of thought whose energies have always been concentrated on clinical observations solely.

Among the ardent upholders of the diatheses as recognisable entities, none has been more assiduous than Sir Dyce Duckworth. Repeatedly he has lectured on the subject at the Seamen's Hospital post-graduate courses; and in the January number of the *Practitioner* he makes a firm stand for the arthritic or rheumatic diathesis, which he describes in great detail. Unfortunately there are but few items in this description which can be recognised in health; they are nearly all morbid conditions the occurrence of which is held to prove that the patient exhibiting them belongs to the arthritic diathesis. In early years, says Sir Dyce, there is often no appearance of any delicacy; and the general development is satisfactory. Dilatation of the facial capillaries is, however, one of the points by which the future arthritic can be recognised; and a languid circulation in the small vessels, leading to cold extremities and chilblains, is also mentioned. Apart from this scanty foundation for diagnosis, the discovery of the diathesis depends on the occurrence of various diseases, of which a large number are mentioned. Some of these, such as tonsillitis, rheumatic fever, carditis, septicæmia, are diseases known to be of microbic origin; others, such as gout, cyclical albuminuria, indigestion, neuralgia, piles, diabetes, and gall-stones, are either of indeterminate or multiple causation.

With all respect to the eminence and experience of Sir Dyce Duckworth, it must be submitted that he is doing exactly what has brought discredit on other diathetic zealots, namely, arguing in a circle. For him all the lesions just mentioned (and others which we have no space to mention) are the evidences of the arthritic diathesis; and the arthritic diathesis is, by definition, the tendency to these diseases. That there is some antagonism between gout and tuberculosis, in the sense that the latter is rare among the subjects of the former, may well be the case, though even that simple proposition might not survive the searching investigation of Professor Karl Pearson's school; but that this is proof of two antagonistic diatheses, the arthritic and the tuberculous, is a generalisation which cannot be supported on such a slender thread of proof. It is at least consoling to note, however, that the whole world is not necessarily either of scrofulous, arthritic, bilious or nervous habit; for Sir Dyke Duckworth grants that there are many whose textures and metabolic processes appear to be normal and void of special proclivities to definite morbid conditions. When he can teach us how to distinguish before the onset of disease between these and the subjects of his four diatheses, the subject may be usefully reconsidered; but until then it is unlikely that he will carry with him the concurrence of the present generation of medical practitioners.

